# Male mating season range expansion results from an increase in scale of daily movements for a polygynous–promiscuous bird

**DOI:** 10.1002/ece3.11302

**Published:** 2024-04-25

**Authors:** Holly Lott, Erin E. Ulrey, John C. Kilgo, Bret A. Collier, Michael J. Chamberlain, Michael E. Byrne

**Affiliations:** ^1^ School of Natural Resources University of Missouri Columbia Missouri USA; ^2^ Warnell School of Forestry and Natural Resources University of Georgia Athens Georgia USA; ^3^ United States Department of Agriculture Forest Service Southern Research Station New Ellenton South Carolina USA; ^4^ School of Renewable Natural Resources Louisiana State University Baton Rouge Louisiana USA; ^5^ Present address: Tall Timbers Research Station Tallahassee Florida USA; ^6^ Present address: NOAA Great Lakes Environmental Research Laboratory Ann Arbor Michigan USA

**Keywords:** animal movement, home range, *Meleagris gallopavo*, space use, telemetry, wild turkey

## Abstract

Males of species with promiscuous mating systems are commonly observed to use larger ranges during the mating season relative to non‐mating seasons, which is often attributed to a change in movements related to reproductive activities. However, few studies link seasonal range sizes to variation in daily space use patterns to provide insight into the behavioral mechanisms underlying mating season range expansion. We studied 20 GPS‐tagged male wild turkeys (*Meleagris gallopavo*), a large upland gamebird, during the mating and summer non‐mating seasons to test the hypothesis that larger mating season ranges resulted from male wild turkeys expanding the scale of daily movement activities to locate and court females. We delineated mating and non‐mating seasons based on intensity of gobbling, a vocalization tied to courtship behavior, recorded by autonomous recording units distributed across the study area. Mating season ranges were significantly larger than non‐mating season ranges. Daily ranges were larger in the mating season, as were distances between roost sites used on consecutive nights. Variance in daily range size was greater in the mating season, but low temporal autocorrelation suggested considerable daily variability in both seasons. We found no evidence that male wild turkeys changed how they distributed daily movements within seasonal ranges, or differences in habitat use, suggesting larger mating season ranges result from male wild turkeys increasing the scale of their daily movements, rather than a systematic shift to a nomadic movement strategy. Likely, the distribution of females is more dynamic and ephemeral compared to other resources, prompting males to traverse larger daily ranges during the mating season to locate and court females. Our work illustrates the utility of using daily movement to understand the behavioral process underlying larger space use patterns.

## Graphical abstract

Males of species with promiscuous mating systems are commonly observed to use larger ranges during the mating season relative to non‐mating seasons, but few studies link seasonal range sizes to variation in daily space use patterns to provide insight into the behavioral mechanisms underlying mating season range expansion. We studied GPS‐tagged male wild turkeys during the mating and summer non‐mating seasons and found larger mating season ranges result from male wild turkeys increasing the scale of their daily movements, rather than a systematic shift to a nomadic movement strategy.

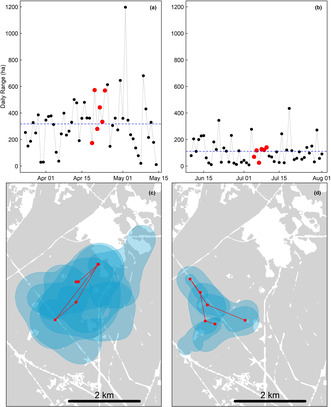

## 1 INTRODUCTION

Understanding animal space use is an important aspect of wildlife science, providing insight into ecology and informing conservation and management. Space use is often considered synonymous with the range of an animal, commonly conceptualized as the area used within a given time frame, which in telemetry‐based studies is often delineated as the area within the 95% or 99% probability contour of a utilization distribution (UD; Worton, 1989). Observed ranges emerge from the accumulated movement responses of an animal over time to dynamic variables such as distribution of resources, interactions with conspecifics, and intrinsic factors such as sex and reproductive state (Börger et al., 2008; Horne et al., 2008; Viana et al., 2018). It is common in wildlife studies to compare range sizes estimated on scales of months or seasons; however, aggregating tracking data over broad temporal scales or generalized seasonal delineations can obscure underlying behavioral patterns of space use that give rise to such ranges (Kie et al., 2010). For example, an increase in range size between seasons could result from an expansion of the average daily area traversed, or by maintaining a consistent daily range size but regularly switching activity areas while tracking changes in resource availability (Lenz et al., 2015). Furthermore, generalized seasonal delineations may encompass, and thus mask, a range of distinct phenological behaviors, or alternatively, such behaviors may bleed across artificial seasonal boundaries making ecological interpretation difficult (Moscicki et al., 2022). Leveraging space use and movement behaviors on short temporal scales such as days or weeks, and recognizing biologically meaningful phenological periods, should allow biologists to infer ecological and behavioral processes that give rise to broader space use patterns (Börger et al., 2006; Kie et al., 2010; Lenz et al., 2015; Moscicki et al., 2022).

The wild turkey (*Meleagris gallopavo*; hereafter, turkey) is a large galliform found throughout much of North America and is an important game species across much of its range (Chamberlain et al., 2022). Turkeys use a polygynous–promiscuous mating system where males compete with conspecifics for breeding opportunities and engage in courtship displays to attract females during the spring mating season, but do not participate in subsequent nest or offspring care (Healy, 1992). Thus, females are an important, widely dispersed, and dynamic resource for male turkeys during the mating season. Following mating season, male behavior shifts from locating and courting females to a maintenance state focused primarily on foraging (Byrne et al., 2015; Holdstock et al., 2006). It is reasonable to hypothesize that the shift in resource priorities from mating to foraging should affect daily movement strategies and consequently result in different space use patterns between mating and non‐mating seasons. During the mating season, males may need to regularly traverse large daily ranges to maximize encounters with receptive females, resulting in relatively large mating season ranges. Increased movement to locate mates is often cited as a mechanism explaining male ranges observed during seasons that encompass mating activities in other polygynous–promiscuous species (Attuquayefio et al., 1986; Byrne & Chamberlain, 2011; Dahle & Swenson, 2003). However, during the summer non‐mating season, when food resources may be more temporally and spatially predictable than females are during the mating season, males may be able to traverse smaller daily ranges to meet their needs (Roth & Vetter, 2008).

Previous research offers some support for male turkey range expansion concomitant with spring mating activities. In South Carolina, USA, males tracked via GPS telemetry during March–May exhibited peaks in mean daily distance traveled during April and May (Chamberlain et al., 2018; Collier et al., 2017), and across southeastern North America, a peak in distance between roost sites used on consecutive nights occurred in April (Byrne et al., 2015). However, properly ascribing changes in movement and ranging behavior to mating activities requires accurately delineating mating and non‐mating seasons. While previous studies have compared movements and seasonal ranges of male turkeys, they have relied on seasonal boundaries delineated based on generalized phenological timings to define, for example, a spring and summer season, with each season encompassing 3–4 months (Badyaev et al., 1996; Godwin et al., 1994, 1995; Grisham et al., 2008; Kelley et al., 1988; Miller et al., 1997). The spring season in these studies ostensibly encompasses mating activities but is long enough to potentially include pre‐ and post‐mating behavioral states as well, which may partially explain conflicting results among studies, with some studies reporting larger spring ranges (Godwin et al., 1995; Kelley et al., 1988) and others reporting no significant difference between spring and summer ranges (Badyaev et al., 1996, Grisham et al., 2008, Miller et al., 1997). Hunting represents an additional potentially confounding variable, as male turkeys are primarily hunted during the mating season, and hunting disturbance and removal of individuals may affect male movement behaviors (Gross, Cohen, et al., 2015; Wakefield et al., 2020; Wightman et al., 2019; Wightman, Martin, Kohl, Collier, & Chamberlain, 2023; Wightman, Martin, Kohl, Rushton, et al., 2023).

We monitored adult male turkeys via GPS telemetry during the mating and summer non‐mating seasons to make inferences on how changes in seasonal range sizes can be attributed to changes in the scale and distribution of daily movement behavior. We conducted research on a non‐hunted population to avoid potential confounding effects of hunting disturbance and used autonomous recording units (ARUs) to delineate mating and non‐mating seasons based on intensity of gobbling. Gobbling is a vocalization used by males to maintain dominance and attract females for mating (Healy, 1992), often elicited in response to female vocalizations (Scott & Boeker, 1972), increased testosterone during the mating season (Lisano & Kennamer, 1977), and female receptivity (Miller et al., 1997; Norman et al., 2001). Because gobbling is associated with courtship behaviors, it provides a means to objectively identify mating season timing specific to a given year and study area. By removing the potentially confounding effects of hunter disturbance and delineating seasons based on direct quantification of a behavior associated with courtship activity, we can make inferences on the role of reproductive activities on changes in space use.

We quantified seasonal range sizes and several metrics of daily space use related to scale and distribution of movements to test the hypothesis that mating season ranges are larger than non‐mating season ranges because courtship and mate‐searching activities require males to expand the scale of daily movements. Consequently, we predicted daily ranges would be larger during the mating season, as searching for females and visiting multiple display sites should require males to traverse large portions of their annual range (Chamberlain et al., 2018; Godwin et al., 1994). We also predicted males would use more widely dispersed roost locations during the mating season, resulting in greater average distances between roost locations used on consecutive nights (Byrne et al., 2015). We predict males expand their range in the mating season by expanding the scale of daily movements while maintaining a consistent home range‐centric movement strategy (Bakner et al., 2022; Mueller & Fagan, 2008). However, it is plausible that seasonal range expansion may instead result from the adoption of a more nomadic movement strategy during mate searching, where males consistently change the location, rather than the size, of daily ranges (Lenz et al., 2015). As such, we quantified patterns of daily location dispersion and aggregation to provide insight into how wild turkeys distributed daily movements within their seasonal ranges and to identify whether wild turkeys shifted daily movement strategies between seasons.

## 2 MATERIALS AND METHODS

### 2.1 Study site

We conducted research on the Savannah River Site (SRS), a 78,000‐ha U.S. Department of Energy property located in the Atlantic Coastal plain of South Carolina, USA. Approximately 94% of SRS was forested, dominated by loblolly pine (*Pinus taeda*), longleaf pine (*P. palustris*), and slash pine (*P. elliottii*) managed by the U.S. Department of Agriculture Forest Service for timber production and wildlife habitat, particularly the federally endangered red‐cockaded woodpecker (*Leuconotopicus borealis*), which involved use of prescribed fire applied on a 3–5‐year rotation (White & Gaines, 2000). Remaining forest cover consisted primarily of bottomland hardwood forests along drainages and the Savannah River. Interspersed within the forest matrix were several nuclear industrial and research facilities, with non‐developed openings consisting of powerline rights‐of‐way and wildlife food plots. Since 1951, there was limited hunting pressure on SRS. The only wild turkey hunting occurred during an annual 2‐day event in late April for 15–25 mobility‐impaired hunters with an average of 25–40 wild turkeys harvested, restricted to limited portions of the property, but hunting was suspended during the years of our study because of the SARS COVID‐19 pandemic.

### 2.2 Tagging and telemetry

During January–March 2020 and December–March 2021, we captured male wild turkeys using rocket nets at sites baited with corn within forest openings. We aged individuals using the presence of barring on the ninth and tenth primary feathers to identify birds >1 year old (Pelham and Dickson 1992). We banded all birds with a numbered aluminum leg band and fitted adults with a backpack‐style GPS transmitter (Guthrie et al., 2011; Lotek Wireless, Newmarket, ON, Canada) programmed to record locations hourly from 0500 to 2000 and one roost location at 2359 until the transmitter battery died or the transmitter was recovered following a mortality (Cohen et al., 2018). We monitored wild turkeys 2–3 times per week with a 3‐element handheld antenna and receiver and remotely downloaded GPS locations approximately once per week via UHF/VHF link to a handheld base station from March 1 to August 1. All capture and handling was approved under the University of Georgia Institutional Animal Care and Use Protocol A2020 06‐018‐R1.

### 2.3 Mating season identification

We collected gobbling data via ARUs (SM3 and SM4, Wildlife Acoustics, Maynard, MA, USA) deployed from March 1 to June 30 annually. We placed ARUs approximately 3 m above ground on trunks of pine trees to minimize potential animal interference. We attached a microphone to the ARU and placed it on the trunk of the tree at a height of 6–9 m to increase sampling range (Colbert et al., 2015; Wightman et al., 2019). We deployed 30 ARUs at sites where wild turkey activity was previously observed. We placed units >600 m apart to prevent gobbles from being recorded on multiple units simultaneously (Colbert et al., 2015). We programmed units to record from 0500 to 1000 in 2020 and 0500 to 1100 in 2021 as most gobbling occurred within 2 hours of sunrise (Wightman et al., 2019). Recordings were analyzed using a convolutional neural network (CNN) with a reported mean precision of 0.32 to identify possible gobbling events (Wightman et al., 2022), which were then manually evaluated to remove false positives.

For each year, we used Poisson regression fit via generalized additive mixed‐effects models (GAMM) to model the number of gobbles as a function of day of the year using the ‘mgcv’ package (Wood, 2017) in R (R Core Team, 2023). We included a random effect for individual ARU to account for variation among units resulting from uneven distribution of gobbling across the landscape. We examined fitted models and delineated mating season boundaries to encompass time periods in each year when gobbling activity was greatest, which we defined as the dates bounding the first and second peaks in gobbling activity, when gobbles per day were greater than one‐half the difference between the greatest expected gobbles per day and the lowest expected gobbles per day preceding the first peak. We delineated non‐mating season as time periods following mating season with <1 predicted gobble per day. Mating activity does not abruptly stop in wild turkeys, but rather tapers off and ends at different times for different individuals (Chamberlain et al., 2018; Healy, 1992; Scott & Boeker, 1972). To account for asynchrony in mating activity timing within the population, we identified a transition period between the mating and non‐mating seasons characterized by a rapid decline in gobbles per day following the mating season peak, which we excluded from analysis to ensure that data in each season was representative of mating and non‐mating behaviors, respectively. Likewise, we attempted to set season boundaries in each year that resulted in mating and non‐mating seasons being of similar lengths.

### 2.4 Daily and seasonal ranges

To estimate daily and seasonal ranges, we used dynamic Brownian Bridge Movement Models (dBBMMs), which allow estimation of a UD conditioned on an animal's movement path while accounting for telemetry error and heterogeneity in behavior (Kranstauber et al., 2012). Following Byrne et al. (2019), we estimated daily ranges for each individual by first fitting a dBBMM to the full movement path using the ‘move’ package (Kranstauber et al., 2023) in R (R Core Team, 2023) to estimate the Brownian motion variance between each set of GPS locations. We then estimated daily UDs on a 5‐m^2^ resolution spatial grid based on all GPS locations and the estimated Brownian motion variance between locations, for all movements recorded between sunrise and sunset each day. We quantified daily ranges as the area within the 99% UD contour as an estimate of the total area used by a wild turkey in a day. We excluded days with <5 GPS fixes (Cohen et al., 2018). We quantified mating and non‐mating season ranges as the area within the 99% UD contour estimated from all movements within the mating and non‐mating seasonal boundaries in each year.

To test whether mating season ranges were larger than non‐mating season ranges, we used a generalized linear mixed‐effects model (GLMM) with a gamma link function that included season as a categorical fixed effect (reference category = mating season) and a random effect (intercept) for individual wild turkeys to account for individual variation. We used a similar GLMM model to test whether daily ranges were larger during the mating season, with season included as a fixed effect and individual included as a random effect. We used a GLMM to test for seasonal differences in distance between consecutive nightly roost locations by including season as a categorical fixed effect and individual turkeys as a random effect. We used a gamma distribution link function for all GLMMs analyzing range size and distances between roosts because these data were continuous, non‐negative, and right skewed. For each respective analysis, we considered seasonal range sizes, daily range sizes, and distances between consecutive nightly roosts to be significantly different between seasons if the 95% confidence interval of the parameter estimate for the fixed effect of season did not cross 0.

We calculated variance in daily range size for each turkey and season and log‐transformed variance to normalize distributions. To test for seasonal differences in variance, we used a linear mixed‐effects model with season as a categorical fixed effect and turkey ID as a random intercept term. To investigate temporal patterns and test whether daily range size was correlated with range size in previous days, we calculated autocorrelation functions in time series of daily range sizes for each male seasonally using the acf function in R (R Core Team, 2023). We determined the number of days where range size was significantly autocorrelated as the number of days with autocorrelation values beyond the 95% confidence bounds for strict white noise.

### 2.5 Patterns of locations within seasonal ranges

To characterize how male turkeys used space within their seasonal ranges, we examined dispersion and aggregation of daily locations relative to all other locations in a season. For each turkey, we treated GPS locations within each day and all other GPS locations within a season, as two parts of a multivariate point pattern. We calculated the difference in intensity of aggregation between each part as the difference in the univariate K‐functions, which estimate the expected density of locations within a distance, *r*, around any randomly chosen location, as follows:

$$D\left(r\right)\sim K_{1}\left(r\right)-K_{2}\left(r\right)$$

where $K_{2}\left(r\right)$ is the function for the daily point pattern and $K_{1}\left(r\right)$ is the function for the point pattern of all other GPS locations in that season (Dixon, 2002). We calculated aggregation intensity daily for each turkey across a range of distances (*r*) in 1 m increments starting from *r* = 1 m using the ‘K1K2’ function in the ‘ecespa’ package (De la Cruz Rot et al., 2010) in R (R Core Team, 2023) and then calculated the seasonal mean at each distance for each individual. We determined a maximum value of *r* based on observed movement capacity individually for each turkey seasonally as the median extent in the *x* or *y* dimension (whichever was greater) of the distribution of each daily set of GPS locations. Aggregation intensity values near 0 indicate daily locations are well dispersed throughout a seasonal range, whereas increasingly negative values indicate daily locations are increasingly spatially aggregated within a seasonal range. Mean values within a season that are highly aggregated suggest adoption of a nomadic movement strategy in which a turkey regularly concentrated daily movements within relatively small portions of its seasonal range and did not often revisit locations used in previous days (Lenz et al., 2015; Mueller et al., 2011). Conversely, a consistent pattern of dispersion (mean values near 0) suggests a turkey consistently revisited large portions of its seasonal range. Our goal was to determine whether there was a meaningful shift in behavior between seasons; thus, our focus was not on interpreting absolute values of *D*(*r*), but rather the relative differences between seasons. We plotted mean seasonal aggregation values for all turkeys to compare relative differences in daily ranging behavior between seasons. If turkeys shifted behaviors seasonally, we would expect to see large and consistent separation in mean value curves between seasons. Additionally, we calculated the difference in mean aggregation intensity between seasons for individual turkeys to illustrate relative magnitudes of change at the individual level. Values >0 suggest greater mean aggregation of daily locations during the mating season, whereas values <0 suggest greater mean aggregation of daily locations during the non‐mating season.

### 2.6 Habitat use

To account for the possibility that seasonal shifts in space use could be driven by systematic shifts in habitat use, we tested for seasonal differences in the proportion of time spent in forested landcover types and distance to forest edges. Using the 2019 National Landcover Database (NLCD; https://www.mrlc.gov/data), which provides landcover data at 30‐m resolution, we categorized landcover into forest (deciduous forest, evergreen forest, mixed forest, and woody wetland NLCD classifications) and non‐forest types. We augmented raw NLCD categorizations by merging NLCD with a shapefile of roads within SRS and categorized roads as open landcover. We felt this was important as personal observations suggested roads were commonly used by males for travel, foraging, and display and not all roads were captured in the NLCD. We classified forest edges as the edge between forest and any open landcover type. To test for differences in use of forest vs. non‐forest landcover types, we used Poisson regression to model the number of GPS locations within forest for each turkey by including season as a categorical fixed effect, including an offset term for the total number of GPS locations collected for each turkey in a season, and including individual as a random effect. We quantified the distance between GPS locations and nearest forest edge and tested for seasonal differences using a GLMM (gamma link function) with season as a categorical fixed effect and individual as a random effect.

## 3 RESULTS

**FIGURE 1:**
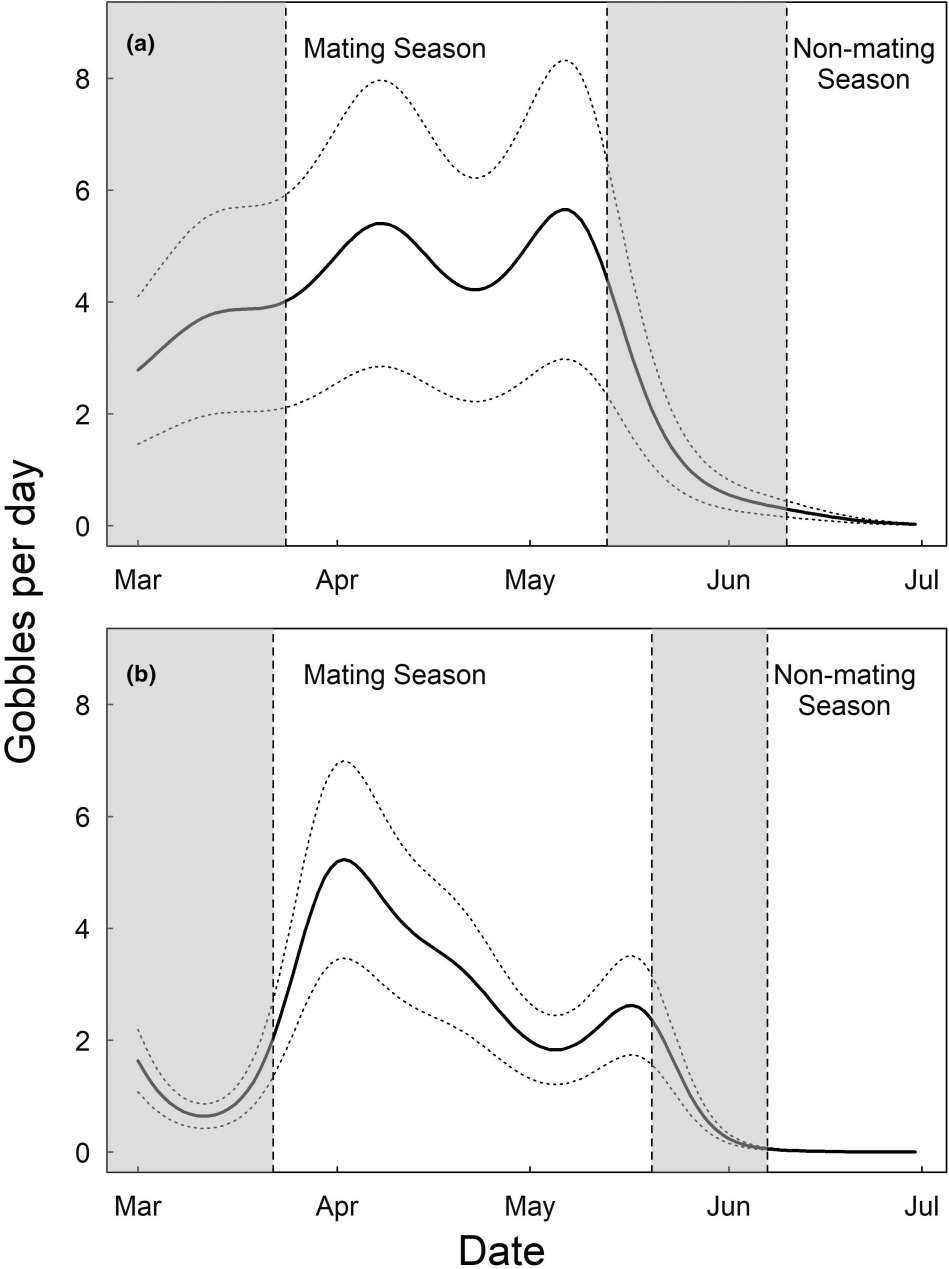
Predicted gobbles per day (with standard error) based on generalized additive models fit to data from autonomous recording devices (*n* = 30) distributed across the Savannah River Site, South Carolina, USA, during spring 2020 (a) and 2021 (b). Vertical dashed lines denote delineations of the mating season and start of the non‐mating season in each year. Shaded areas correspond to pre‐mating and transition periods.

Based on 42,733 confirmed gobbles recorded in 2020, we delineated the mating season as March 24–May 13 (7 weeks) and the non‐mating season as June 10–July 31 (7.5 weeks). Based on 19,590 gobbles recorded in 2021, we set the mating season as March 22− May 20 (7.9 weeks) and the non‐mating season as June 7–July 31 (7.6 weeks; Figure 1). We captured and tagged 10 adult male turkeys in 2020 and 25 adult males in 2021. We excluded individuals that experienced transmitter malfunctions (*n* = 13) or died soon after capture (*n* = 3) from all analyses. Two males died after the mating season and thus did not provide data during the non‐mating season. Overall, we obtained mating season data for 18 males and non‐mating season data for 16 males. After exclusion of days with <5 GPS fixes (*n* = 13), we retained 450 days of mating season data in 2020, 660 days of mating season data in 2021, 464 days of non‐mating season data in 2020, and 605 days of non‐mating season data in 2021.

**FIGURE 2:**
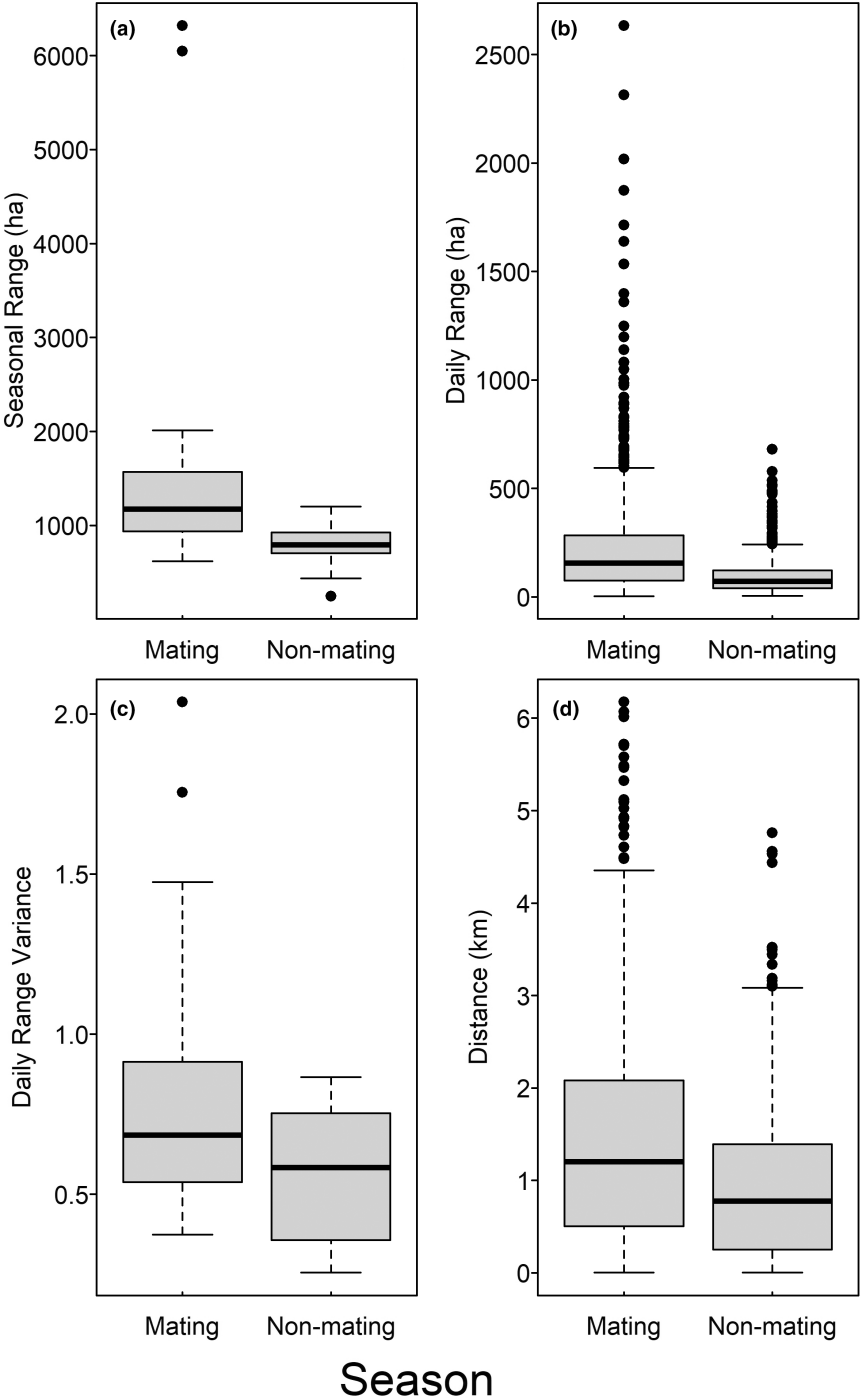
Boxplots representing the distributions of seasonal range size (a), daily range size (b), log variance in daily range size (c), and distance between roost sites used on consecutive nights (d) between the mating and non‐mating seasons for GPS‐tagged male wild turkeys on the Savannah River Site, South Carolina, USA, 2020–2021.

There was a significant difference in seasonal range sizes (Table 1), with mating season ranges (median = 1172 ha, range: 617.5–6319 ha) larger than non‐mating season ranges (median = 791.7 ha, range: 246.6–1202 ha; Figure 2). Most turkeys (83%) with data for both seasons used smaller non‐mating season ranges, with an average decrease in range size of 38.1% (range: 16.7%–86.6%) relative to the mating season. There was a significant decrease in daily range size (Table 1) in the non‐mating season (median = 70.7 ha, range: 4.3–680.5 ha) compared to the mating season (median = 155.3 ha, range: 2.7–2632.9 ha; Figure 2). Two individuals regularly traversed exceptionally large daily mating season ranges, accounting for 52.6% and 42.1% of all daily ranges >800 ha, respectively. Both individuals also maintained the largest mating season ranges. On average, males decreased median daily range size by 50.6% (range: 2.4–76.0%) from the mating season to the non‐mating season. There was a significant seasonal decrease in distance between consecutive nightly roost locations (Table 1) in the non‐mating season (median = 0.77 km, range: 0.002–4.76 km) relative to the mating season (median = 1.20 km, range: 0.002–6.17 km; Figure 2).

**TABLE 1:** Results of mixed‐effects models of male wild turkey space use, distances between roost locations, use of landcover types, and distance from forest edges between mating and non‐mating seasons on the Savannah River Site, South Carolina, USA, 2020–2021.

Response variable	Intercept	Season	Random effect
Seasonal range size	7.1 (6.9 to 7.5)	−0.51 (−0.70 to −0.32)	0.19 (0.43)
Daily range size	5.3 (5.1 to 5.5)	−0.75 (−0.85 to −0.66)	0.11 (0.33)
Variance in daily range size	0.84 (0.67 to 0.99)	−0.26 (−0.46 to −0.06)	0.04 (0.19)
Distance between roosts	0.31 (0.17 to 0.45)	−0.40 (−0.50 to −0.29)	0.06 (0.24)
Distance from forest edge	3.9 (3.7 to 4.1)	−0.001 (−0.02 to 0.02)	0.13 (0.36)
Number of locations in forest	−0.38 (−0.44 to −0.31)	−0.003 (−0.03 to 0.02)	0.02 (0.15)

*Note*: Presented are fixed‐effects regression coefficient estimates (with 95% confidence intervals) for the intercept and effect of season and the variance (with standard deviation) of the random effect term. In all models, the mating season was the reference category and individual turkeys were included as a random intercept. We inferred a significant difference between seasons when the confidence interval for the estimate of season did not cross 0.

**FIGURE 3:**
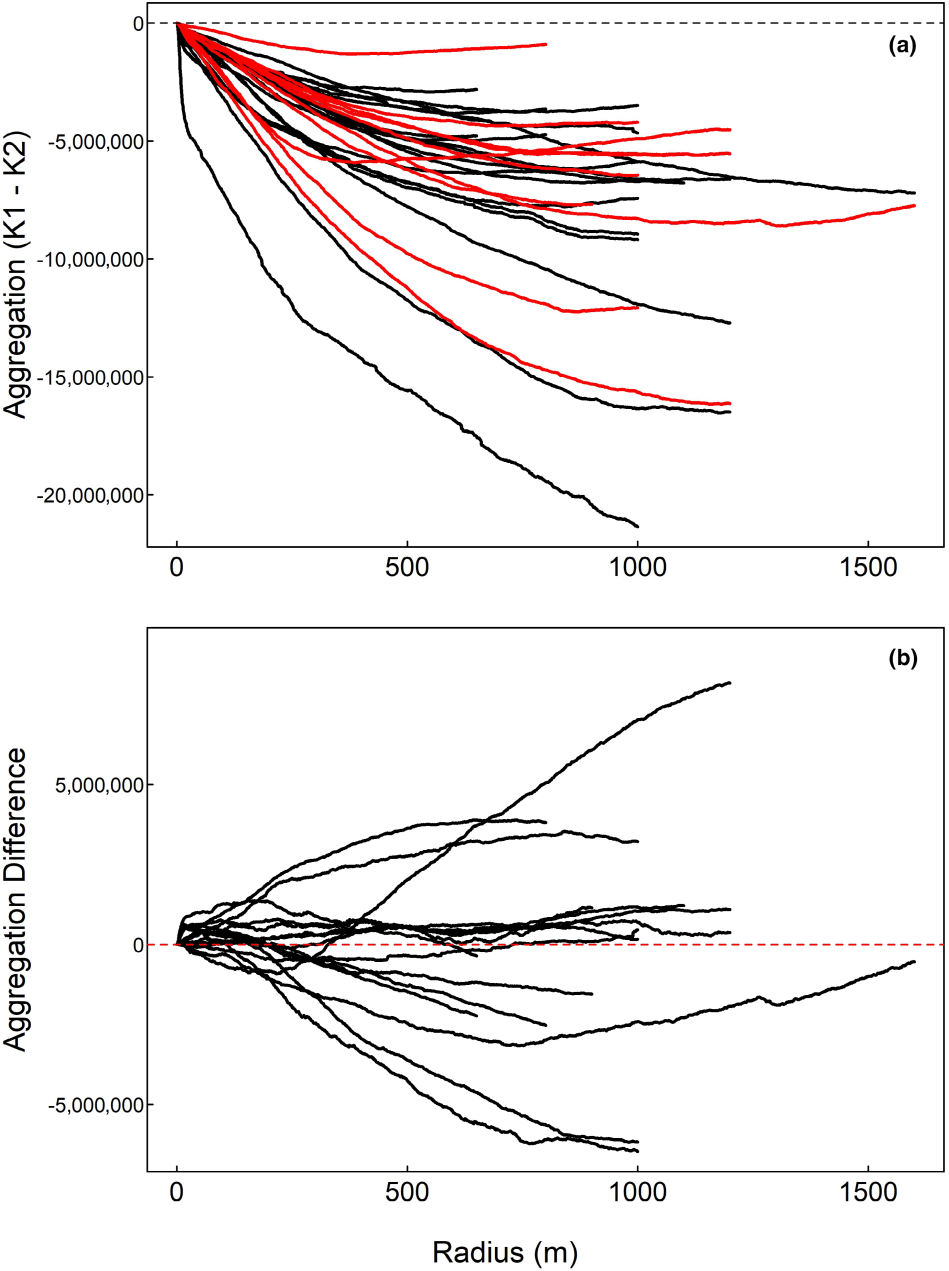
Mean spatial aggregation intensity of daily GPS locations within mating (black) and non‐mating (red) seasonal ranges for 20 GPS‐tagged wild turkeys on the Savannah River Site, South Carolina, USA, 2020–2021 (a). Aggregation intensity values near 0 indicate daily movements are well dispersed throughout a seasonal range, whereas increasingly negative values suggest daily movements occur within relatively small portions of a seasonal range. The difference in aggregation intensity between seasons for individual turkeys is shown in (b). Values >0 indicate a turkey's daily movements were on average more aggregated in the non‐mating season, and values <0 indicate greater aggregation during the mating season.

Male turkeys exhibited significantly less variance in daily range size during the non‐mating season (Table 1; Figure 2). Only one male exhibited significant autocorrelation in daily range size during the mating season, with a significant temporal lag of 2 days (Table 2), indicating considerable day‐to‐day variation in range size during the mating season. During the non‐mating season, six males (33%) exhibited significant autocorrelation in daily range size, but apart from one individual, significant temporal lag was ≤2 days (Table 2), indicating that despite lower overall variance there was still considerable day‐to‐day variation in range size during the non‐mating season as well. Maximum values of *r* used to quantify aggregation intensity for individual turkeys ranged from 650 m to 1600 m in the mating season and 800 m to 1600 m in the non‐mating season. There were no clear seasonal differences in mean aggregation intensity (Figure 3a). When comparing seasonal changes in mean aggregation by individual males, there was considerable variation, with no evidence of a consistent population‐level shift in the distribution of daily locations within seasonal ranges between seasons (Figure 3b).

**TABLE 2:** Temporal autocorrelation of daily range sizes during the mating and non‐mating seasons for GPS‐tagged male wild turkeys on the Savannah River Site, South Carolina, USA, 2020–2021.

Turkey ID	Year	Significantly autocorrelated days	
		Mating	Non‐mating
61241	2020	0	0
61242	2020	2	2
61243	2020	0	1
61289	2020	0	0
61297	2020	0	0
61303	2020	0	0
61308	2020	0	NA
61307	2020	0	0
61311	2020	0	0
47401	2021	0	0
47402	2021	0	1
47403	2021	0	0
47409	2021	0	1
47417	2021	0	0
47418	2021	0	0
47427	2021	0	0
47428	2021	0	0
47441	2021	0	1
47463	2021	0	6
47472	2021	0	NA

*Note*: The above shown are the number of significantly autocorrelated days for each individual turkey and season.

Habitat use was consistent between seasons, with no significant differences in use of forest or distance to forest edge (Table 1). Mean proportion of GPS locations in forest was 0.70 during both mating (range: 0.53–0.86) and non‐mating (range: 0.49–0.90) seasons. The mean proportion of available forest within seasonal ranges was similar in the mating (mean = 0.80, range: 0.69–0.92) and non‐mating (mean = 0.80, range: 0.70–0.91) seasons. Males spent considerable time near forest edges in both seasons, with a median distance of 32 m during the mating season (range: 0–484 m) and 33 m during the non‐mating season (range: 0–419 m).

## 4 DISCUSSION

Our study is unique in that we objectively differentiated mating and non‐mating seasons based on gobbling intensity on our study site, a behavior directly related to male wild turkey reproductive activities. Additionally, by studying a non‐hunted population, we controlled for potentially confounding effects of hunting on male behavior (Gross, Cohen, et al., 2015; Wakefield et al., 2020; Wightman et al., 2019; Wightman, Martin, Kohl, Collier, & Chamberlain, 2023; Wightman, Martin, Kohl, Rushton, et al., 2023). This allowed us to accurately characterize and compare space use between two phenologically distinct seasons and facilitated making inferences on how seasonal changes in behavioral state (mating vs. non‐mating) and resources (females and food) influenced space use. Thus, we can reasonably infer that seasonal changes in space use we observed can be largely attributed to the response to seasonal changes in reproductive behavior and resource distribution.

Our findings largely supported our hypothesis that reproductive activity leads male turkeys to use larger daily ranges during the mating season, resulting in larger mating season ranges relative to the non‐mating season. Furthermore, there was little evidence that the decrease in range size observed in the non‐mating season resulted from a shift in habitat use. Notably, in both seasons turkeys were strongly associated with forest edges, and despite changes in seasonal range size, the proportion of forest available within seasonal ranges was consistent between mating and non‐mating seasons. Wild turkeys are habitat generalists that use both open and forested landcover types (Byrne et al., 2014; Hall et al., 2007; Miller et al., 2000); thus, it stands to reason that turkeys would often be located at the interfaces between landcover types in both seasons. Additionally, many forest openings on our study area consisted of linear roads and rights‐of‐way, which we commonly observed turkeys using as travel corridors in both seasons. A consistent shift in aggregation intensity of daily locations between seasons could indicate male turkeys exhibited population‐level behavioral shifts as observed in male trumpeter hornbills (*Bycanistes bucinator*), whose winter range expansion relative to the breeding season was associated with a shift toward a nomadic movement strategy of exploiting a profitable forage patch for several days before moving on (Lenz et al., 2015). However, we observed similar levels of daily aggregation intensity between seasons and considerable individual variability, with male turkeys exhibiting both increased and decreased aggregation intensity in the non‐mating season (Figure 3). The lack of a consistent seasonal signal in aggregation intensity of daily locations suggests that like habitat use, a biologically significant shift in how turkeys distributed daily movements within seasonal ranges does not explain differences in seasonal range sizes.

**FIGURE 4:**
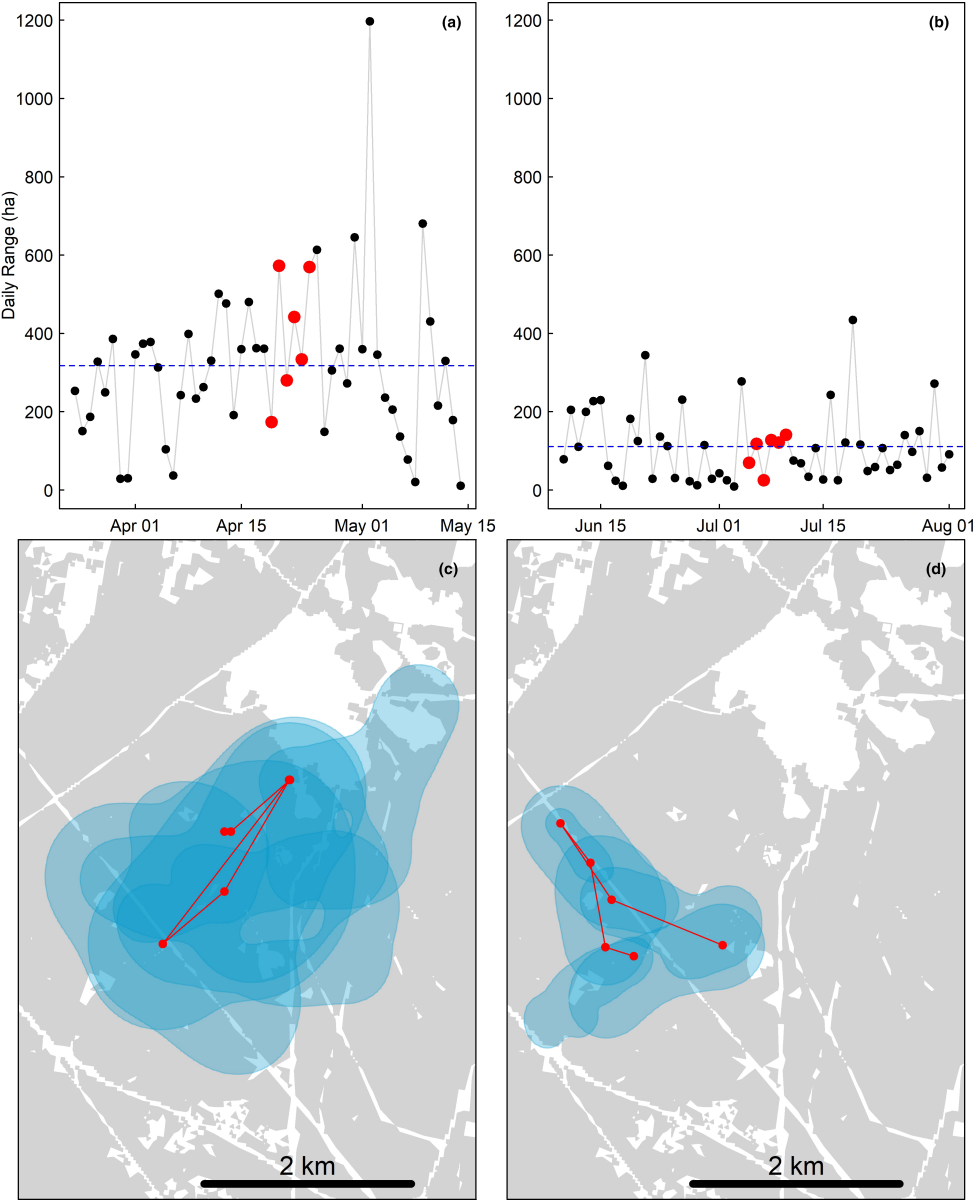
Time series of daily range size during the mating (a) and non‐mating (b) seasons for a representative GPS‐tagged male wild turkey on the Savannah River Site, South Carolina, USA, in 2020. Horizontal dashed lines represent mean values. Red dots in panel (a) correspond to plotted daily ranges in panel (c), and red dots in panel (b) correspond to plotted ranges in panel (d). Red locations in panels (c, d) are roost locations for days plotted. Gray represented forest landcover in panels (c, d).

Our results suggest that rather than the adoption of a different movement strategy or change in habitat use, the large seasonal range sizes of male turkeys in the mating season result from a general increase in scale of daily movements, as evidenced by greater daily range sizes and distance between nightly roost locations (Figure 4). Previous studies (Chamberlain et al., 2018; Collier et al., 2017; Godwin et al., 1994) have suggested that like males of other promiscuous species, increased daily movements of male turkeys in spring are a consequence of reproductive activities (Attuquayefio et al., 1986; Byrne & Chamberlain, 2011; Dahle & Swenson, 2003; Long et al., 2013). After partitioning seasons based on gobbling activity and without confounding effects of hunting disturbance, our results support this hypothesis, as all turkeys in our study used larger median daily ranges during the mating season. During the mating season, females represent a dynamic and patchily distributed resource and maximizing encounters with receptive females likely requires male turkeys to regularly traverse large portions of their home ranges, resulting in larger average daily ranges. During the non‐mating season, foraging resources are likely more abundant and predictable than females are during the mating season, and as males are no longer searching for females, they likely do not need to regularly traverse similarly large areas. Additionally, use of widely dispersed roost locations may help maximize opportunities of encountering receptive females during the mating season (Byrne et al., 2015; Wakefield et al., 2020), as males frequently call from roosts, which provide elevated locations that reduce predation risk and maximize sound propagation (Boncoraglio & Saino, 2007; Ey & Fischer, 2009; Mathevon et al., 2005).

Turkeys exhibit considerable daily fluctuations in testosterone levels (Bacon et al., 1991), which is known to lead to daily fluctuation in reproductive behaviors in other Galliformes (Wada, 1986). Chamberlain et al. (2018) suggested that fluctuating testosterone levels partially explained daily variation in gobbling activity, and we suspect such fluctuations play a role in daily variations in movement as well. In this context, the large variance in daily range size observed in the mating season suggests a general pattern of days engaged in reproductive behaviors such as courtship and mate searching, interspersed with days characterized by a reduction in reproductive activities. However, we recognize that it is impossible to draw direct correlative inference between daily range size and engagement in reproductive activities, as it is possible that males may not always need to traverse large areas to encounter females, and other factors such as weather and encounters with predators may also influence behavior (Cresswell, 2008; McMillan et al., 2022; Rivrud et al., 2010). Minimal temporal autocorrelation in range size across both seasons suggests that despite lower absolute variance in daily range size during the non‐mating season, there was still considerable day‐to‐day variability (Figure 4). This finding is consistent with the evidence that turkeys distribute daily movements similarly within seasonal ranges in both seasons (Figure 3) and further supports the suggestion that the decrease in range sizes during the non‐mating season is a consequence of a general decrease in scale of movements rather than a change in overall movement strategy.

Our study site was heavily forested, and our results may not translate directly to populations in regions dominated by open landcover types. For example, landscapes with minimal forest cover and reduced roost‐site availability may place limitations on daily movements and range sizes that turkeys may not face in a heavily forested landscape such as SRS, where suitable roost sites are ubiquitous (Byrne et al., 2015; Gross, Little, et al., 2015). Additionally, variations in courtship behavior have been observed between subspecies of wild turkeys living in different landscape types (Healy, 1992). Male turkeys in forested landscapes such as SRS display individually or in small groups at widely dispersed locations comparable to exploded leks (Ulrey et al., 2023), but there have been observations of males in grassland‐dominated landscapes forming true leks, communal display grounds visited by females in which males display in larger groups (Healy, 1992; Watts & Stokes, 1971). Differences in the distribution and availability of resources and suitable habitats across landscapes likely result in differences in seasonal space use patterns of male turkeys. Similar studies across a range of landscapes would provide opportunities to assess the relative role of context‐dependent habitat and reproductive activity on male turkey space use.

Our study illustrates the need for delineating biologically meaningful time periods to understand changes in space use behavior. Moscicki et al. (2022) showed how generalized, arbitrary seasonal delineation can mask important phenological variation associated with different reproductive phases for female turkeys. If we were to rely on generalized seasonal delineation of mating and non‐mating seasons commonly applied to studies of male turkeys (Badyaev et al., 1996; Godwin et al., 1994, 1995; Grisham et al., 2008; Kelley et al., 1988; Miller et al., 1997), it would have made ecological interpretation difficult. A generally defined 3‐month reproductive season of March–May, for example, would have included mating activities each year, but also included periods of pre‐ and post‐mating activities. Our study also illustrates the advantages of using daily movements to understand the behavioral processes influencing seasonal space use patterns. In our case, we were able to make biological inferences on how different daily movement patterns during the mating season resulted in larger seasonal ranges. Other studies have leveraged space use data across multiple temporal scales to make inferences on how environmental conditions and behavioral changes give rise to observations on annual or seasonal scales (Börger et al., 2006; Lenz et al., 2015; Rivrud et al., 2010; Viana et al., 2018). Given the increasing ubiquity of GPS and other tracking technologies that provide fine‐scale location data over long time periods in modern wildlife ecology, more studies could harness such data to answer interesting biological questions about space use behavior (Fieberg & Borger, 2012; Kie et al., 2010).

## AUTHOR CONTRIBUTIONS

**Holly Lott:** Data curation (equal); formal analysis (equal); investigation (equal); methodology (equal); writing – original draft (lead); writing – review and editing (equal). **Erin E. Ulrey:** Investigation (equal); writing – review and editing (equal). **John C. Kilgo:** Resources (equal); writing – review and editing (equal). **Bret A. Collier:** Conceptualization (equal); funding acquisition (equal); resources (equal); writing – review and editing (equal). **Michael J. Chamberlain:** Conceptualization (equal); funding acquisition (equal); resources (equal); writing – review and editing (equal). **Michael E. Byrne:** Conceptualization (equal); data curation (equal); resources (equal); writing – review and editing (equal).

## CONFLICT OF INTEREST STATEMENT

The authors declare no conflict of interest.

## Data Availability

Wild turkey GPS tracking data are available in the Dryad online data repository: https://doi.org/10.5061/dryad.rv15dv4fb.
